# Increased mortality for colorectal cancer patients with preexisting diabetes mellitus: an updated meta-analysis

**DOI:** 10.18632/oncotarget.19923

**Published:** 2017-08-04

**Authors:** Jingtao Li, Jixi Liu, Chun Gao, Fang Liu, Hongchuan Zhao

**Affiliations:** ^1^ Department of Gastroenterology, China-Japan Friendship Hospital, Beijing, China

**Keywords:** diabetes mellitus, colorectal cancer, survival, mortality, meta-analysis

## Abstract

**Background:**

Although the preexisting diabetes mellitus (DM) is known to have a high risk for death in many cancers, its impact on the mortality for the colorectal cancer (CRC) patients is still uncertain. In this study, we conducted a meta-analysis to explore an association of DM with the survival for the CRC patients.

**Materials and Methods:**

We made a relative data search from the public available databases including Medline and Embase with a cutoff date to Jan 31, 2017. Pooled hazard ratios (HRs) were calculated using either a fixed or random effect model. Trim and fill analysis was conducted to test and adjust for publication bias. Subgroup analyses were also performed for overall survival and all-cause mortality when stratified by tumor stage, geographical region, duration of follow-up, gender and subsite of cancer.

**Results:**

Twenty-one eligible cohorts including 1,025,034 patients were identified and included in this meta-analysis review. The sample size for each analysis was ranged from 207 to 771,297 patients. It is revealed that with the preexisting DM, the CRC patients had a significantly increased all-cause mortality (pooled adjusted HR: 1.23; 95% CI: 1.11, 1.37) and decreased overall survival (pooled adjusted HR: 1.25, 95% CI: 1.19–1.31). But no difference was found for adjusted cancer-specific survival for the CRC patients with the preexisting DM compared with subjects without DM. These associations almost remained consistent after trim and fill adjustment and across those outcomes when stratified by site of cancer, tumor stage, population geography, study design, duration of follow-up, data resource or gender.

**Conclusions:**

This meta-analysis review indicates that preexisting diabetes mellitus in CRC patients is severely associated with the worse overall survival but not with cancer-specific survival.

## INTRODUCTION

As the third most frequently diagnosed cancer through the worldwide, colorectal cancer (CRC) is the fourth leading cause and account for around 8% of all cancer-related death [1]. There are 95,520 new cases of colorectal cancer in 2017 in the United States [2]. The colorectal cancer is also known to affects men and women almost equally [3], the colorectal cancer represents about 9.4% of all incident cancer in men and 10.1% in women [4].

The colorectal cancer survival is severely dependent on the stage of disease at diagnosis. 90% for the 5-year survival rate for the cancer detected at the localized stage; 70% for the regional, 10% for the people diagnosed as distant metastatic cancer [5, 6]. Meanwhile, the colorectal cancer may be influenced by some risk factors. For instance, the colorectal cancer is distributed severely dependent on the region not uniformly through the world [7]. In fact, it has been demonstrated that the colorectal cancer displays a large geographic difference through the global distribution. In addition, the incidence of colorectal cancer is known to highly rely upon some personal factors as well as other diseases for the patients such as age, personal history of adenomatous polyps, and inflammatory bowel disease, family history of colorectal cancer or adenomatous polyps, inherited genetic risk as well as factors from the environment [5–7].

Diabetes mellitus (DM) is a group of common metabolic and endocrine diseases, and is known as one of the greatest threats to the human health through the worldwide [8]. As an independent risk factor for the CRC prognosis, DM has been explored in several early reports to observe its influence to the survival of CRC patients on the basis of meta-analysis [9, 10]. However, because of limited data searches and analytical strategies, the early reports cannot offer sufficient evidence supporting the effect of preexisting DM on the survival for the CRC patients [11]. In addition, to our knowledge, there is lack of a report regarding the subgroup analyses from the geographic region to the subsite of cancer on this subject.

We are interested in using meta-analysis model to explore the impact of preexisting DM on the survival of CRC patients. In recent years, there were several publications appearing on the cohorts of CRC patients with the preexisting DM [12–13], which might provide us a great opportunity to determine the role of DM on the progression of CRC. In this report, the data achieved from these new cohorts as well as from early reports were used to conduct a meta-analysis on the impact of preexisting DM on the survival of CRC patient subgroup analyses from the geographic region to the subsite of cancer. We predict the results in this review might provide us a scientific implication on the impact of preexisting DM to the survival of CRC patients and furthermore a scientific guidance to the potential clinical applications [13].

## MATERIALS AND METHODS

### Literature search strategies

Literatures were searched by two independent investigators listed as primary authors in this article (J Li and J Liu) and from the databases of Medline (since January 1, 1966) and Embase (since January 1, 1974) through the due time of Jan 31, 2017 without any language restriction. Medical subject heading (MeSH) terms and keywords for our search strategies are: diabetes or diabetes mellitus or NIDDM; neoplasm or cancer or adenocarcinoma or carcinoma or tumor or tumour; colon or colorectal or rectal; survival or mortality or prognosis or outcome. The reference lists were also reviewed to identify additional relevant studies. The complete search strategy was shown in the supplementary data (Supplementary Document 1). Eligible records were input into an Endnote library.

### Inclusion and exclusion criteria

The cases included in this analysis must meet the following 4 criteria: 1) participants: colorectal patients, 2) intervention and comparisons: DM versus Non-DM, 3) outcomes: available hazard ratios (HRs) with corresponding 95% of Confidence Intervals (CIs) of overall survival (OS) or having sufficient information to reconstruct them, and 4) study design: cohort study. Diabetes status was obtained from the patients’ self-report questionnaires, blood glucose test information, medical records, and study reports in the articles.

Some cases which meet the criteria were excluded in our analysis only when: 1) without sufficient or consistent data; 2) the mortality of patients being reported in hospital or after surgery; and 3) inadequate information for evaluating the diabetes associated HR and 95% CIs.

### Data extraction and quality assessment

Data extraction was performed by two independent investigators in this analysis (J Li and J Liu), and verified independently for the accuracy by the third investigator (C Gao). For each eligible case, the information of first author, publication year, location, study design, date of recruitments, median follow-up, sample size, number of DM/Non-DM patients, number of death for the DM/Non-DM patients, study population source, DM ascertainment, adjustments variables, crude and adjusted all-cause HRs and 95% CIs, and cancer-specific HRs and 95% CIs were independently extracted in the form of piloted structure. Any disagreement during the case selection or data collection was resolved by consensus among three authors of J Li, J Liu, and C Gao as referred back to the original articles.

The quality of each cohort study was assessed with developed star system (ranging from 0 to 9 stars) of Newcastle-Ottowa Scale (NOS) [14]. At this scale, the studies were evaluated with three categories: selection with 4 stars and comparability with 2 stars for the study groups, and assessment of outcome with 3 stars for the interest. The quality was scored with a star rating system from 0 to 9, in which 0–5 stars represent a low quality while 6–9 stars represent a high quality [15].

### Statistical analysis

Adjusted estimation was achieved by precedence for a quantitative analysis, Missing or incomplete estimation and 95% CIs were used in the calculation for achieving the appropriate summary statistics or Kaplan-Meier curves [16]. HRs and corresponding 95% CIs were used to calculate the overall and cancer-specific survival. Heterogeneity among the analyses was assessed using Cochrane *Q* test with a significance level of *p* ≤ 0.1, and quantified by estimated *I*^2^ with a value of > 50% as the standard of significant heterogeneity [17].

Several methods were used to test a potential publication bias including a primary strategy of visual inspection of funnel plot, Begg’s test and Egger’s test [18]. In addition, we conducted a trim and fill analysis to test and adjust for publication bias. The subgroups analyses were performed on the basis of cancer subsites of colorectal cancer, colon cancer, and rectal cancer. In detail, we discussed the influences from the TNM staging, geographical region, median follow-up time, and sex of patients.

Sensitivity analyses were employed to validate the robustness of strategy according to the adjusted HRs with 95% CI. A fixed effect model was used to estimate the pooled HR with 95% CIs if there was no evidence of heterogeneity; otherwise, a random effect model was employed.

All *p* values were obtained from *two*-*tailed tests*. Statistical analyses were performed using Stata SE version 12.0 software (StataCorp, College Station, TX, USA) and Review Manager Version 5.3 software (Nordic Cochrane Centre in Copenhagen of Denmark, 2014).

## RESULTS

### Literature search and study characteristics

**Figure 1:**
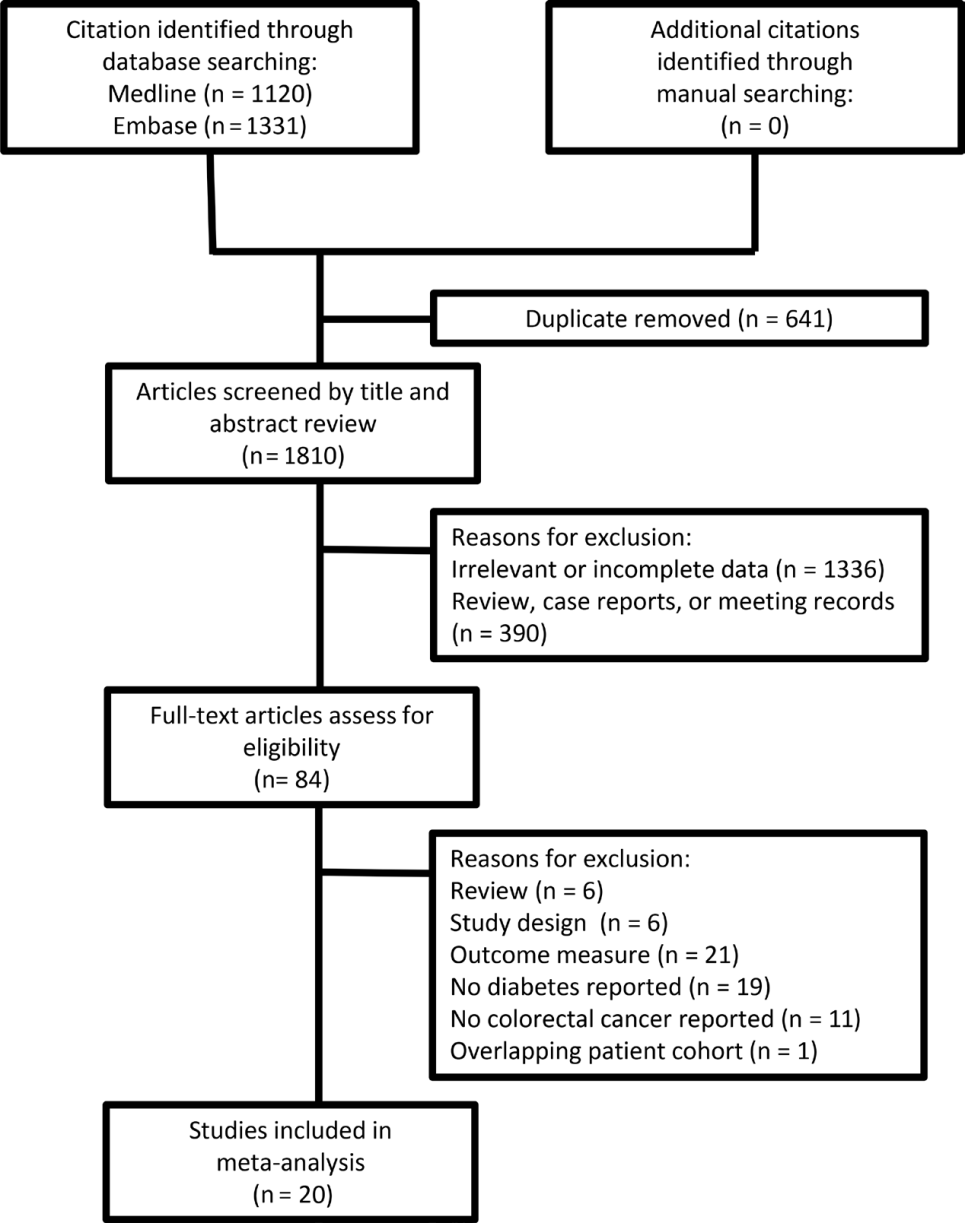
Flowchart for the selection of study cases in the meta-analyses

The flow diagram for the case selection is presented in Figure 1. In the search of dataset, 21 cohorts were found to enable satisfying the criteria what we set for the analysis strategy, and thus, include in this study [12, 13, 19–36]. The characteristics of cohort cases in this review were listed in Supplementary Table 1. The sample size was ranged from 207 to 771,297 and the median follow-up was ranged from 2.67–15.6 years [12, 36].

From 2,451 titles and abstracts identified in the literature search, 84 full articles were subjected to make a further review because of their eligibility, and 64 articles were decided to exclude due to at least one of following reasons: overlapping of patients [37], lack of definition for DM or CRC, and/or lack of focus on OS. Finally, 20 articles were identified for the current analysis to investigate the association of preexisting DM with the overall survival of CRC patient (Supplementary Table 1), including 10 prospective studies [13, 19–27] and 10 retrospective case studies [12, 28–36]. In addition, the report from van de Poll-Franse LV et al. was classified into two groups of colon cancer and rectal cancer because two diseases were reported in this article [33]. The colon cancer and rectal cancer cases were also noticed to unevenly select in which there were only three cases enrolled into the colon cancer [22, 25, 26] and all others into the rectal cancer.

HRs of all 16 cohorts could be used to achieve adjusted overall survival, 5 cohorts for adjusted cancer-specific survival and 9 for all-cause mortality.

### Meta-analysis on DM and overall survival or all-cause mortality

**Figure 2:**
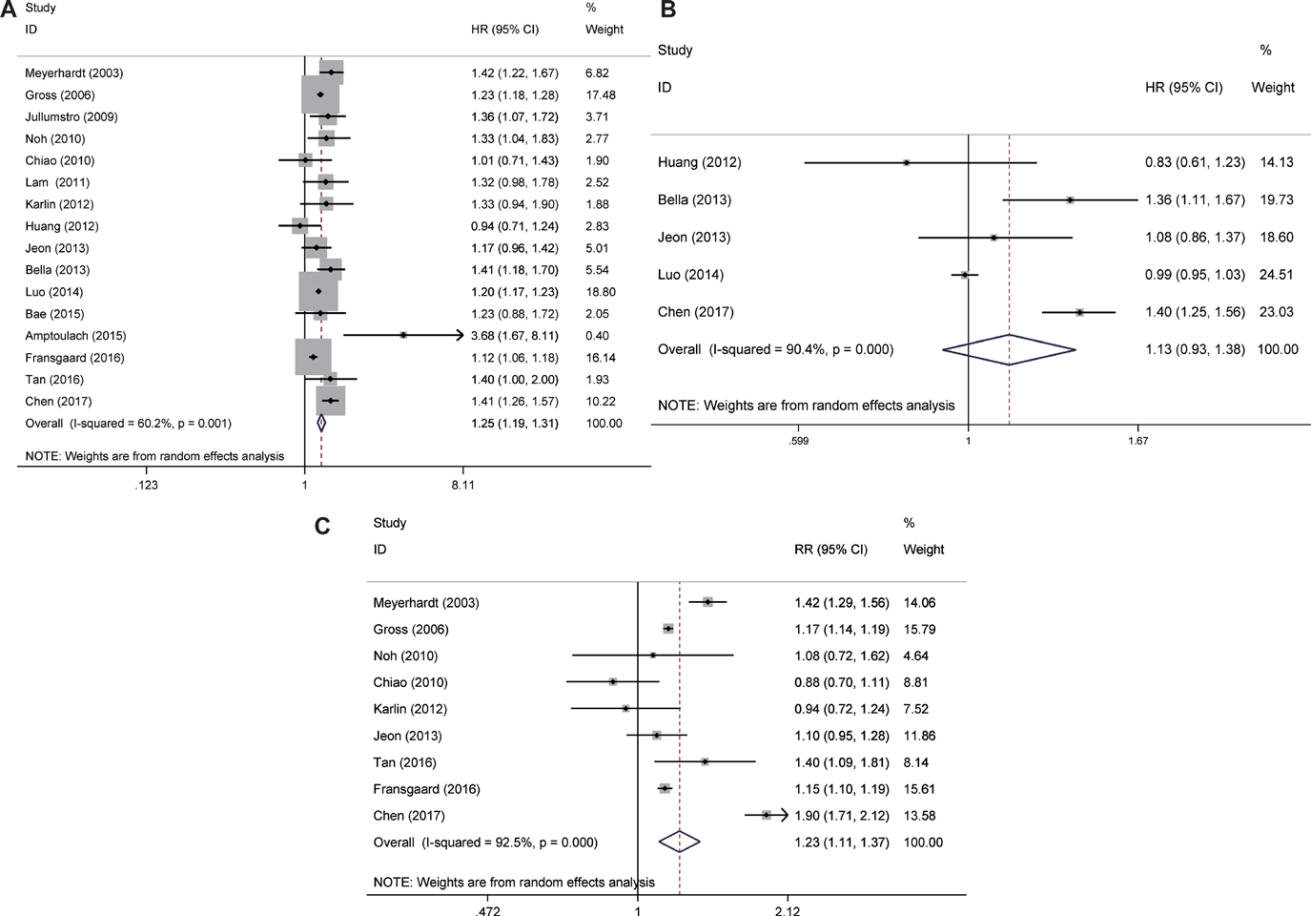
Meta-analysis of the main outcomes of colorectal cancer (**A**) adjusted overall survival, (**B**) adjusted cancer-specific survival, (**C**) all-cause mortality. Abbreviations: HR, hazard ratio; CI, confidence intervals.

Large variations on the covariates were arisen for the data from these simply enrolled reports. So pooled HR for adjusted overall survival was estimated in sixteen enrolled, including 10 cases were found to associate with the survival [12, 13, 19, 24, 27–29, 31, 34, 35], and the values of other cases did not display any statistical significance [21, 23, 26, 30, 32, 36] (Figure 2A). It is revealed that with the preexisting DM, the CRC patients had a significantly decreased overall survival (pooled adjusted HR: 1.25, 95% CI: 1.19–1.31) (Figure 2A).

Pooled HR for all-cause mortality was estimated in 9 studies and shown the increased risk for CRC patients with DM when compared with subjects without DM (pooled HR: 1.23; 95% CI: 1.11, 1.37) (Figure 2C).

Five cases were found to be able to provide HRs of cancer-specific survival for the CRC patients with the DM values. No association was found for adjusted cancer-specific survival for the CRC patients with the preexisting DM (pooled adjusted HR: 1.13; 95% CI: 0.93, 1.38) (Figure 2B).

These associations almost remained consistent after trim and fill adjustment and across those outcomes when stratified by site of cancer (Table 1).

**Table 1:** Meta-analysis of DM and patient mortality outcomes, analyses of publication bias with different models for overall survival, all-cause mortality, cancer-specific survival

**Variable**	**No. of studies**	**HR (95% CI)**	**Heterogenicity**	**Heterogenicity**	**Effect size**	**Effect size**^a^
			***P***^†^	***I***^2^ **(%)**	***Z***	***P***^‡^
**colorectal cancer**						
overall survival	16	**1.247 (1.185–1.312)**	0.001	60.2	**8.53**	**< 0.001**
cancer-specific survival	5	1.132 (0.925–1.384)	< 0.001	90.4	1.20	0.283
all-cause mortality*	9	**1.233 (1.111–1.367)**	< 0.001	92.5	**3.96**	**< 0.001**
**colon cancer**						
overall survival	8	1.222 (1.164–1.283)	0.271	20	8.05	**< 0.001**
cancer-specific survival	6	1.081 (0.991–1.178)	0.098	46.1	1.76	0.078
**rectal cancer**						
overall survival	5	1.237 (1.076–1.421)	0.06	55.8	3	**0.003**
cancer-specific survival	5	1.100 (0.899–1.346)	0.02	65.7	0.92	0.355
Publication bias	Begg’s *p* values	Egger’s *p* values	number of studies added by trim and fill method		T&F(Fill)	
**colorectal cancer**						
overall survival	0.893	0.135	4		**1.198 (1.135–1.263)**	
cancer-specific survival	0.806	0.413	2		0.983 (0.809–1.194)	
all-cause mortality*	0.754	0.547	0		**1.233 (1.112–1.367)**	
**colon cancer**						
overall survival	0.266	0.211	3		**1.196 (1.166–1.227)**	
cancer-specific survival	0.26	0.01	3		1.016 (0.930–1.110)	
**rectal cancer**						
overall survival	0.806	0.77	0		**1.237 (1.076–1.421)**	
cancer-specific survival	0.806	0.764	0		1.100 (0.899–1.346)	

*effect size is Relative Risk.

^†^Two-sided test for heterogeneity (*I*^2^).

^‡^Two-sided test for pooled analysis (*Z* test).

T&F: result of trimmed and filled analysis, using assumption of random effects;

Fill: number of studies added by trim and fill method.

^a^:Significant *P* values (< 0.05) are in boldface.

### Subgroup analysis

The mortality of CRC patients with the preexisting DM is suggested to strongly associate with subsite of cancer [3–7], and thus, subgroup meta-analyses of colorectal cancer were performed to achieve HRs with different strategies.

**Figure 3:**
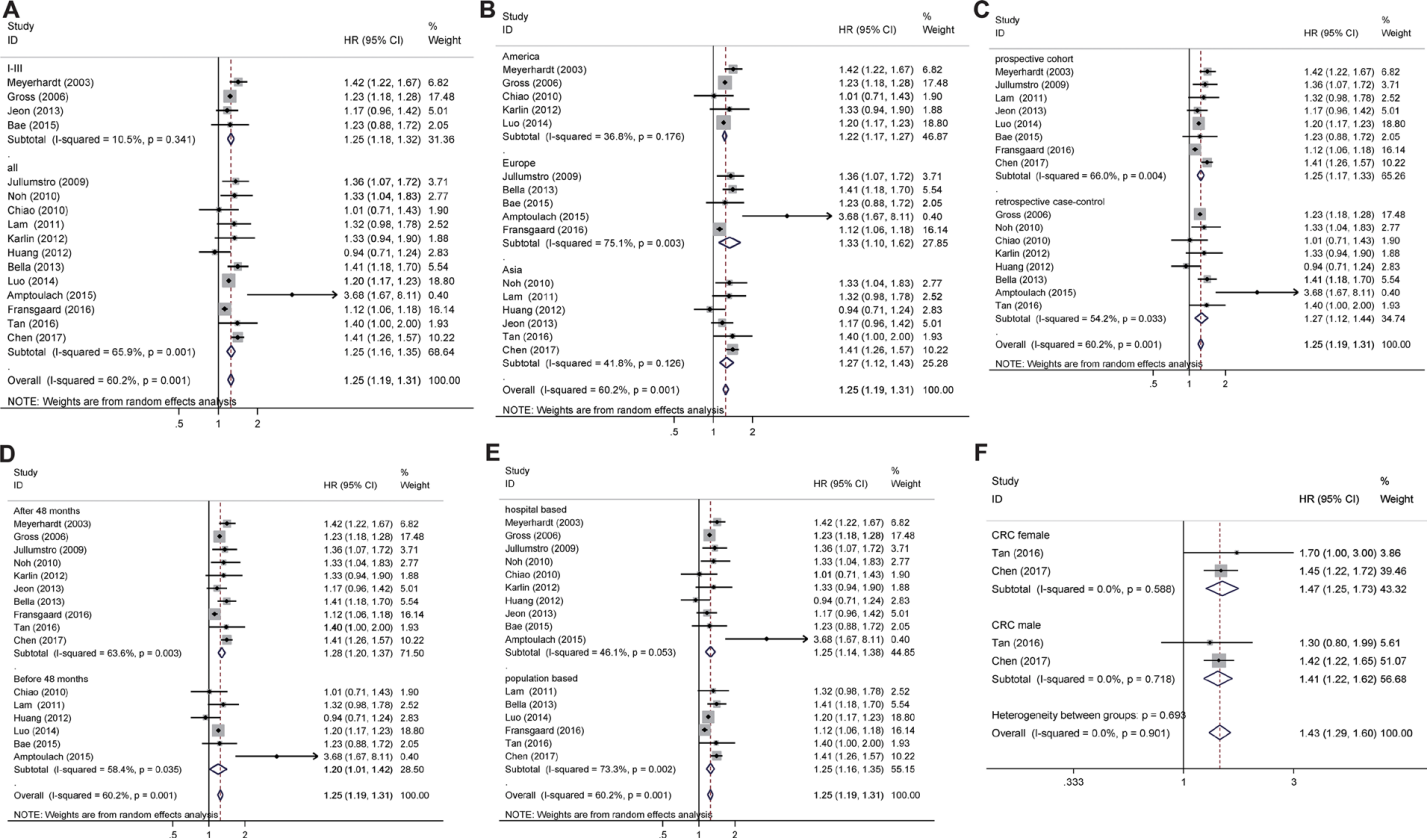
Subgroup analysis for adjusted overall survival (**A**) TNM staging, (**B**) geographical region, (**C**) prospective cohort study and retrospective case-control study, (**D**) follow-up time, (**E**) Source of patient population, and (**F**) sex of patients.

**Figure 4:**
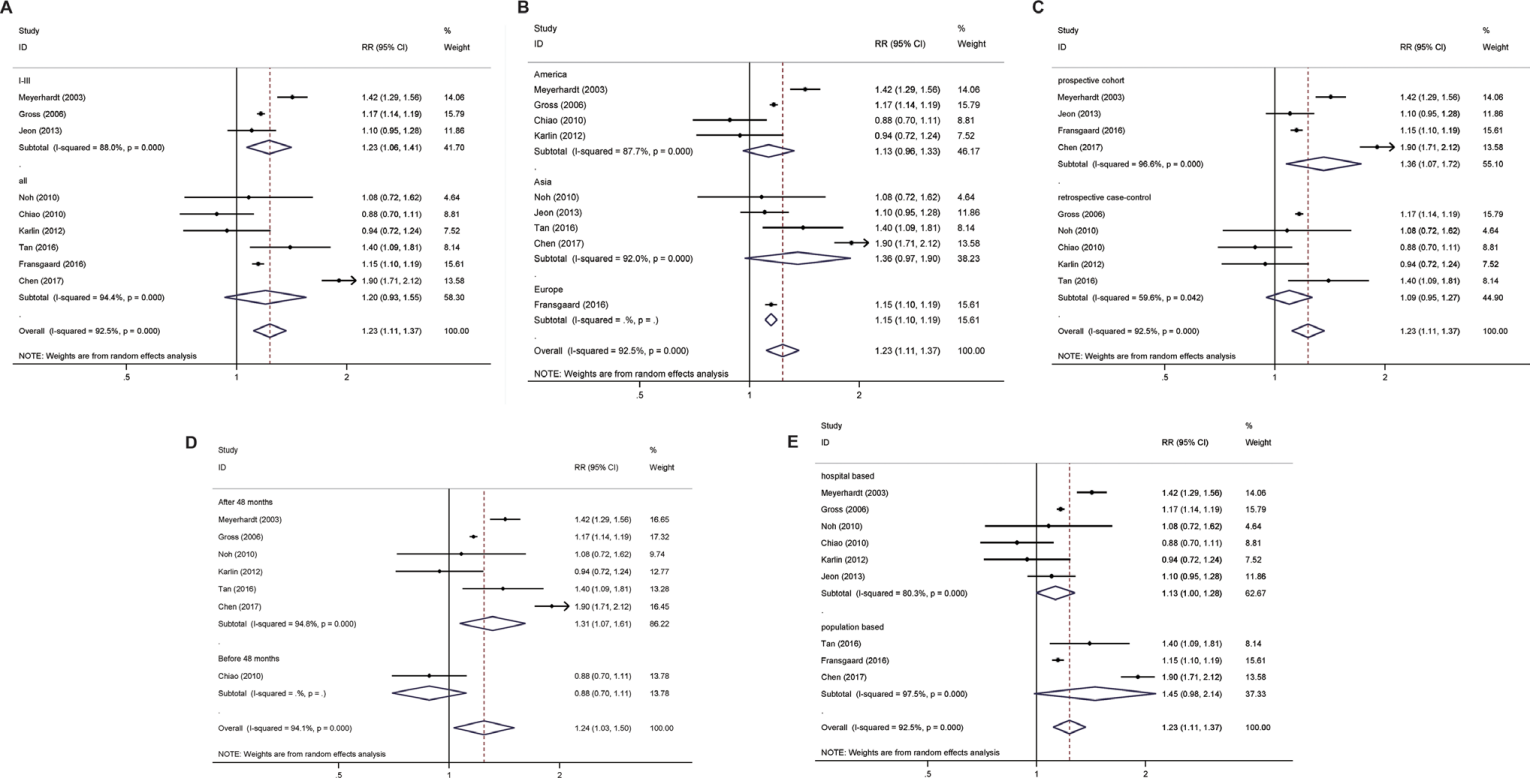
Subgroup analysis for all-cause mortality (**A**) TNM staging, (**B**) geographical region, (**C**) prospective cohort study and retrospective case-control study, (**D**) follow-up time, and (**E**) Source of patient population.

Adjusted overall survival (Figure 3) and all-cause mortality (Figure 4) were calculated and presented in the subgroup subject (Table 2). These associations for overall survival almost remained consistent across the outcomes in all CRC patients when stratified by site of cancer, tumor stage, population geography, study design, duration of follow-up, data resource or gender (Figure 3, Table 2).

**Table 2:** Subgroup analysis of the main outcomes of colorectal cancer

Subgroup	Number of Studies	Heterogeneity		**Model**^#^	Meta-analysis	
		*P*	***I***^2^ **(%)**		HR (95% CI)	***P* for HR**^a^
**Overall survival**						
I–III TNM staging	4	0.341	10.5	Fixed	**1.246 (1.177–1.319)**	**< 0.001**
All TNM staging	12	0.001	65.9	Random	**1.253 ( 1.164–1.348)**	**< 0.001**
North America	5	0.176	36.8	Fixed	**1.223 ( 1.175–1.272)**	**< 0.001**
Europe	5	0.003	75.1	Random	**1.335 (1.100–1.619)**	**0.003**
Asia	6	0.126	41.8	Random	**1.266 (1.120–1.431)**	**< 0.001**
Cohort study	8	0.004	66.0	Random	**1.247 (1.167–1.332)**	**< 0.001**
Retrospective case-control study	8	0.033	54.2	Random	**1.266 (1.115–1.436)**	**< 0.001**
After 48 months follow-up	10	0.003	63.6	Random	**1.281 (1.196–1.372)**	**< 0.001**
Before 48 months follow-up	6	0.035	58.4	Random	**1.197 ( 1.012–1.416)**	**0.036**
Hospital based	10	0.053	46.1	Random	**1.253 (1.141–1.377)**	**< 0.001**
Population source	6	0.002	73.3	Random	**1.251 (1.160–1.349)**	**< 0.001**
All-cause mortality*						
I–III TNM staging	3	< 0.001	88.0	Random	1.226 (1.064–1.413)	**0.005**
All TNM staging	6	< 0.001	94.4	Random	1.199 (0.927–1.551)	0.167
North America	4	< 0.001	87.7	Random	1.128 (0.959–1.328)	0.145
Europe	1	-	-	Random	1.146 (1.104–1.189)	**< 0.001**
Asia	4	< 0.001	92.0	Random	1.356 (0.970–1.895)	0.075
prospective cohort study	4	< 0.001	96.6	Random	1.360 (1.072–1.724)	**0.011**
retrospective case-control study	5	0.042	59.6	Random	1.095 (0.946–1.266)	0.224
After 48 months follow-up	6	< 0.001	94.8	Random	1.314 (1.069 -1.615)	**0.010**
Before 48 months follow-up	1	-	-	Random	0.883 (0.700–1.114)	0.294
Hospital based	6	< 0.001	80.3	Random	1.128 (0.998–1.275)	0.055
Population source	3	< 0.001	97.5	Random	1.450 (0.984–2.138)	0.060

^a^Significant *P* values (< 0.05) are in boldface, -: Only one study included.

*Effect size is Relative Risk.

^#^Random is the Random effect model and Fixed is the Fixed effect model.

### Colorectal cancer subgroup analysis

#### TNM staging

Preexisting diabetes mellitus in CRC patients is associated with the higher all-cause mortality in the I-III TNM staging but not in all TNM staging (Figure 3, Table 2).

#### Geographical region

For the analysis based on only one case, the data was removed from the systemic comparison. It was shown that although the overall survivals display statistic difference, Preexisting diabetes mellitus in CRC patients is not associated with the all-cause mortality in both North America and Asian populations (Figure 4, Table 2).

#### Median follow-up time

As discussed above, for the analysis based on only one case, the data was removed from the systemic comparison. It was shown that the overall survival for the patients both before and after 48 months follow-up is significantly decreased in CRC patients with DM when compared CRC without DM (Figure 3, Table 2). The pooled HR was increased for all-cause mortality for population longer than 48 months follow-up but not for population less than 48 months follow-up (Figure 4, Table 2).

### Publication bias

**Figure 5:**
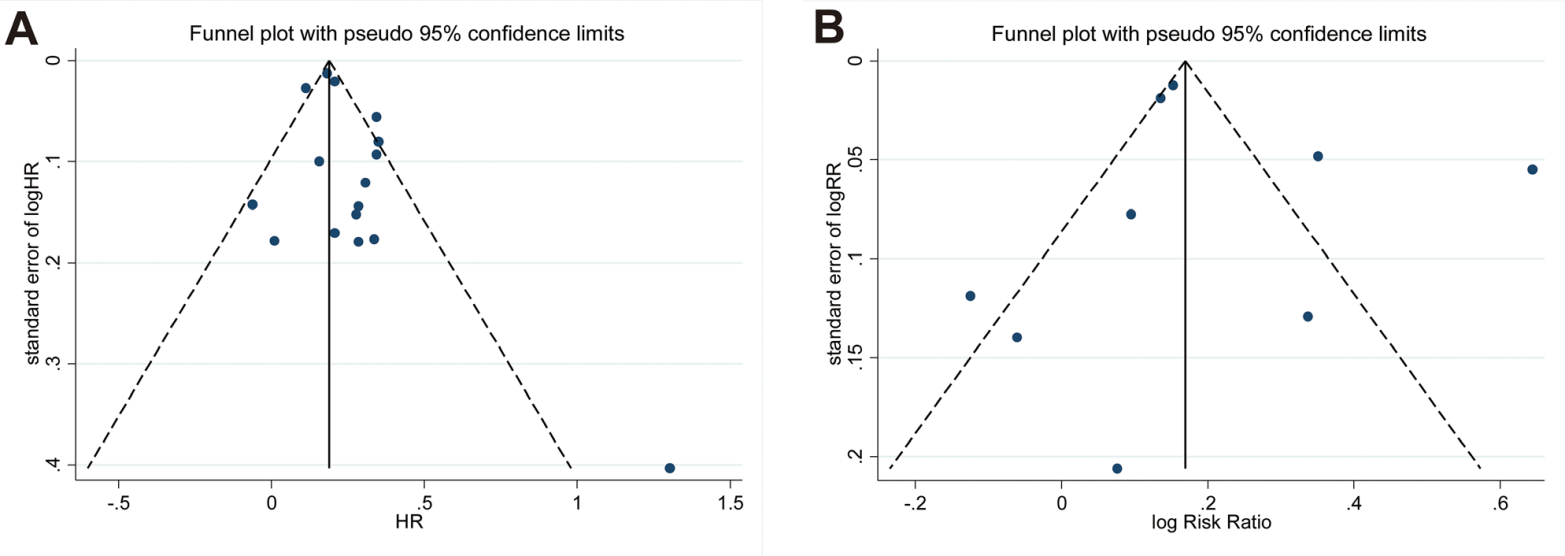
Funnel plot for overall survival and all-cause mortality (**A**) adjusted overall survival and (**B**) all-cause mortality. Abbreviations: HR, hazard ratio.

The publication bias of articles used in the present analysis was evaluated, and there was no asymmetry in the funnel plot among the recurrence of these studies (*p* = 0.135 for adjusted all-cause HR analysis, in Figure 5) by Egger’s test representing no obvious publication bias. An analysis after adjustment using trim and fill method showed decreased pooled HR for overall survival (1.198, 95% CI 1.135–1.263) and increased all-cause mortality (1.233, 95% CI 1.112–1.367) in CRC with DM when compared with CRC without DM (Table 1), approximately matching with the values obtained without any adjustment.

**Figure 6:**
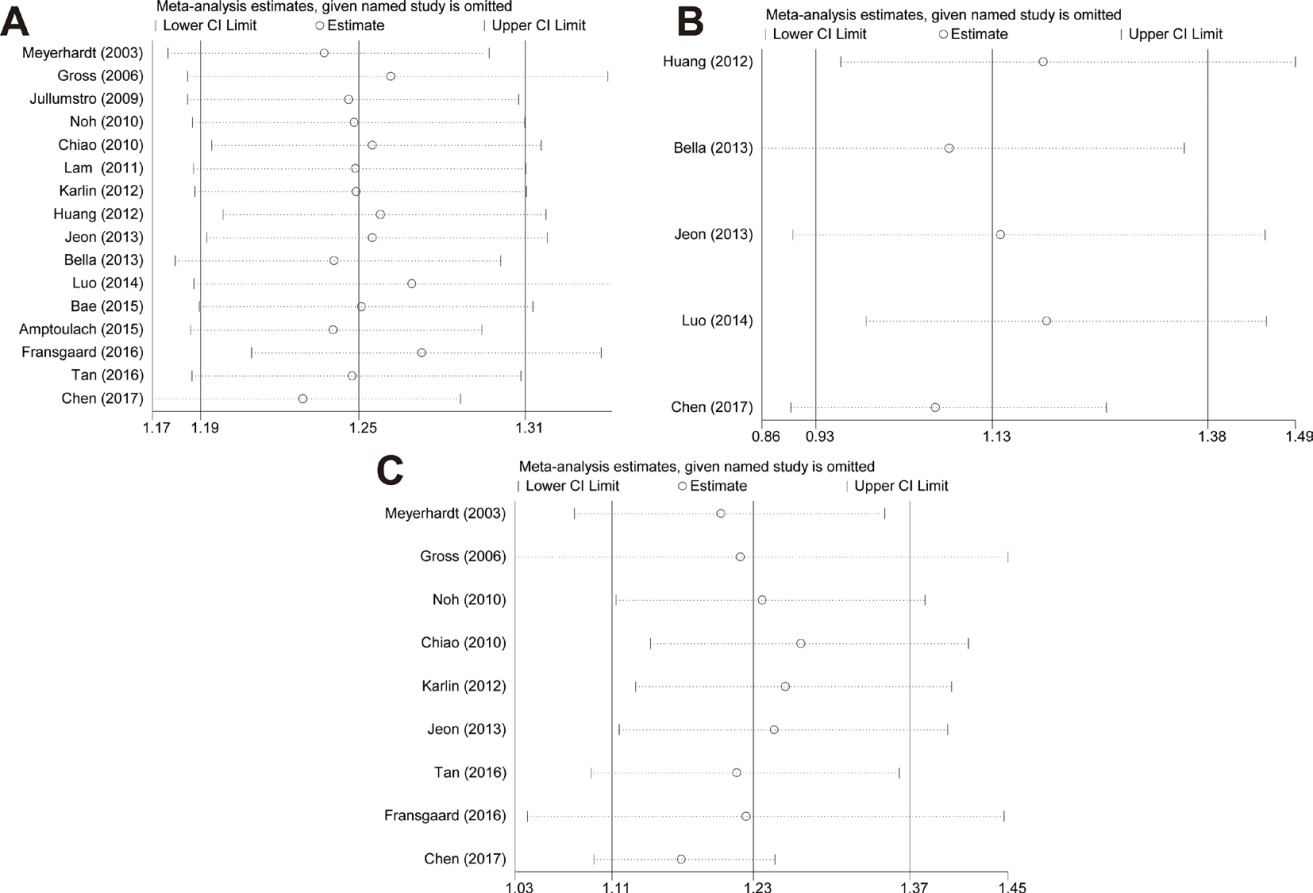
Sensitivity analyses by excluding one study at a time for reflecting the influence of the individual dataset to the pooled HRs (RRs) (**A**) overall survival, (**B**) cancer-specific survival, and (**C**) all-cause mortality.

Sensitivity analyses were used to validate the robustness of strategy according to the crude and adjusted HRs with 95% CI. We performed sensitivity analysis by excluding one study each time and then calculated the pooled HR. It was shown that there was no dramatic change on the trend of pooled data through an exclusion of a specific study (Figure 6).

## DISCUSSION

It is known that the diabetes has a close association with the pancreatic, bladder, and liver cancers [20, 38–43], and as a result, the cancer patients with the preexisting DM are supposed to have relatively higher all-cause mortality. The effect of diabetes on the cancer progress can be also interpreted using insulin and insulin-like growth factor 1(IGF-1) signaling pathways [44]. It is known that increased insulin levels in the body can activate IGF-1 signaling which might furthermore promote the cancer cell proliferation. Accordingly, the IGF-1 receptor has a high expression in some cancer cells, which might in turn receive long-term proliferation signals that are activated by elevated insulin levels. As a result, the cancer recurrence and progression might be increased [43].

The current analysis review indicates that the all-cause mortality is indeed significantly increased for the CRC patients with the preexisting DM. This observation is also found to be approximately consistent with the reports from other meta-analysis groups [7, 8]. In addition, some of other factors, such as age, personal lifestyle, etiology, clinical staging, tumor site and size, and initial treatment, etc., might affect the survival of CRC patients. To avoid the biases from these other factors, the analyses were performed with adjusted and confirmed using trim and fill method. These associations remained consistent after trim and fill adjustment.

It is also noticed that the survival for the CRC patients with the preexisting DM was decreased, which might be due to some causes that are non-cancer related. For example, the DM patients might have other DM-related conditions except the cancer that would affect severely the clinical death. These conditions might include myocardial infarction, cerebrovascular and chronic pulmonary, kidney and peripheral vascular diseases as well as other diseases. The clinical death by all these conditions might influence analytical results in the current study. In addition, the CRC patients with the preexisting DM might lead to a lower sensitivity due to the cancer treatments, as well as higher infection and intraoperative mortality [32, 43]. Thus, in comparison with an all-cause mortality analysis, a cancer-specific mortality analysis is suggested that might reveal the association of DM with the mortality of CRC patients and be more appropriate for this study.

The all-cause mortality was first used to interpret the results in our meta-analysis. In comparison with the non-DM counterparts, the CRC patients with the preexisting DM displayed a 23% increase of all-cause mortality and 25% decrease of overall survival. We also performed the subgroup analyses on the basis of cancer subsites including colorectal cancer, colon cancer, and rectal cancer.

It is found that the HRs of mortality for the colorectal and colon cancer patients are significantly increased with the preexisting DM but almost independent on the staging. In contrast, we also notice that the all-cause mortality of I-III TNM staging is 23% higher but no difference for all TNM staging representing that the preexisting DM on the CRC may influence the survival of patients by TNM staging-dependence.

Comparing three geographical subgroups including North America, Europe, and Asia, HRs of overall survival and cancer-specific survival for the colorectal patients are found to remain almost the same without significant statistic difference.

Even though there are some publications on the single topic of subgroup meta-analysis for the association of mortality for colorectal cancer patients with preexisting diabetes mellitus [8–11], to our knowledge, it is the first time to report the data from the CRC patients with the different subsite and perform subgroup meta-analysis. Among the reasons that cause such subgroup differences, highly expressed IGF-1 by the diabetes for the CRC patients might be regarded as one reason that severely associate with the colorectal cancer risk by the anatomic subsite [45].

We also noted that the death certification information for some CRC patients was unavailable in our literature search. Moreover, the provincial databases were not accessible. All these limitations might limit availability of real cancer-specific mortality in our study. As a result, the analyses on the real cancer-specific mortality were lacking for most analyses. Instead, we conducted a meta-analysis on the DM and cancer-specific mortality using only 5 cases in which the cancer-specific mortality was available. The result revealed that the CRC patients with DM displayed no significant risk on the cancer-specific mortality in comparison with the non-DM counterparts. Further study is needed in the future.

We have to recognize that there were limitations for our meta-analyses in this study which were primarily arisen from the literature searches from several aspects. First, the inclusion criteria, population, and adjustment for the confounding variables are different among the cases that could be avoided in this study. Second, the status of DM duration across the cases was not directly reported in some cases. Third, the effect of therapies on the cancer outcome was not assessed due to absence of information on the anticancer and antidiabetic therapies as well as other related outcomes in most of reports.

On the other hand, the current analytical strategies have been demonstrated to have a large robustness, and as a result, the limitations in the literature search have only insignificant influence on the overall conclusions in this study particularly on the association of DM with increased all-cause mortalities for the CRC patients. We also expect to find an efficient method to overcome these outcomes and further improve the accuracy for our next meta-analysis on the impact of DM to the survival of CRC patients.

## SUPPLEMENTARY MATERIALS TABLE





## Abbreviations

DM: diabetes mellitus
CRC: colorectal cancer
HR: hazard ratio
CIs: Confidence Intervals
OS: overall survival
NOS: Newcastle-Ottowa Scale
MeSH: Medical subject heading
