# Supplementary material for: Increased mortality for colorectal cancer patients with preexisting diabetes mellitus: an updated meta-analysis
Source: Oncotarget. 2017 Aug 4;8(37):62478–88. doi: 10.18632/oncotarget.19923 (PMC5617522; doi:10.18632/oncotarget.19923)
Supplement: Supplementary file 1 [file oncotarget-08-62478-s001.pdf]

Increased mortality for colorectal cancer patients with preexisting diabetes mellitus: an updated meta-analysis

SUPPLEMENTARY MATERIALS

Supplementary Document 1: The complete search strategy

900 Medline (by PUBMED)

((diabetes mellitus[MeSH Terms]) OR (diabetes[Text Word]) OR diabetes mellitus[Text Word]) OR NIDDM[Text Word])) AND ((colorectal cancer[MeSH Terms]) OR (((colon[Text Word]) OR colorectal[Text Word]) OR rectal[Text Word])) AND ((neoplasm*[Text Word]) OR tumor*[Text Word]) OR tumour*[Text Word]) OR cancer*[Text Word]) OR adenocarcinoma*[Text Word]) OR carcinom*[Text Word])) AND ((survival*[Text Word]) OR mortality[Text Word]) OR prognos*[Text Word]) OR outcome*[Text Word])).

1275 Embase

('diabetes mellitus'/exp OR diabetes:tw OR NIDDM:tw) AND ('colorectal cancer'/exp OR colorectral:tw OR colon:tw OR rectal:tw) AND (neoplasm*:tw OR tumor*:tw OR tumour*:tw OR cancer*:tw or adenocarcinoma:tw OR carcinoma:tw) AND (survival*:tw OR mortality:tw OR prognos*:tw OR outcome*:tw).

Supplementary Table 1: Characteristics of 21 Cohorts in the meta-analysis of the effect of preexisting DM on colorectal cancer all-cause mortality. See Supplementary_Table_1
