# Supplementary material for: Increased mortality for colorectal cancer patients with preexisting diabetes mellitus: an updated meta-analysis
Source: Oncotarget. 2017 Aug 4;8(37):62478–88. doi: 10.18632/oncotarget.19923 (PMC5617522; doi:10.18632/oncotarget.19923)
Supplement: Supplementary file 2 [file oncotarget-08-62478-s002.docx]

**Supplementary Table 1: Characteristics of 21 studies included in the meta-analysis of the effect of preexisting DM on colorectal cancer mortality.**

| Study | year | location | Study design | Date of recruitment  (range) | Median Follow-up (yrs) | Sample size | Population source | DM ascertainment | age | adjustments | NOS score |
| --- | --- | --- | --- | --- | --- | --- | --- | --- | --- | --- | --- |
| Meyerhardt JA, et al. ^20^ | 2003 | USA | prospective cohort | 08/1988-07/1992 | 9.4 | 3,549 | hospital based | medical record | 65.5 in male, 61.6 in female | age (50, 50–60, 60–70, 70), body mass index (as continuous variable), gender, race,baseline performance status (0, 1, or 2), bowel obstruction, bowel perforation, stage of disease, presence of peritoneal implants, and completion of chemotherapy. | 8 |
| Gross CP, et al. ^29^ | 2006 | USA | retrospective | 1993-1999 | 4.1 | 29,733 | medicare database | medical record | 77.8 | sociodemographic characteristics, cancer-specific characteristics (stage, histological grade, tumor location), and individual conditions | 8 |
| Jullumstro E, et al. ^30^ | 2009 | Norway | cohort | 1980-2004 | NA | 1,194 | hospital based | registered | 76.2 in DM, 71.7 in non-DM | age, cardiac disease, pulmonary disease, tumor stage | 7 |
| Chen CQ, et al. ^21^ | 2010 | China | prospective cohort | 1994-2002 | 3.8 | 945 | hospital based | medical | 62.3 | tumor stage, intestinal, obstruction, gross type | 7 |
| Chiao EY, et al. ^31^ | 2010 | USA | retrospective cohort | 01/1999-12/2006 | 2.9 | 470 | hospital based | medical | 68 | age, gender, race, year of CRC diagnosis, staging, co-morbidity score, and treatment received | 8 |
| Noh GY, et al. ^32^ | 2010 | Korea | retrospective cohort | 1997-2004 | 4.7 | 657 | hospital based | medical | 61.2 in DM, 57.6 in non-DM | age, BMI | 6 |
| Lam EKK, et al. ^22^ | 2011 | Asia Pacific Region | prospective cohort | 2000-2003 | 4 | NA | population based | Self-reported, blood glucose Levels | NA | age-adjusted, sex and study stratified | 8 |
| Huang YC, et al. ^23^ | 2011 | China | prospective cohort | 01/1999-01/2008 | NA | 2,762 | hospital based | medical | NA | age, gender, stage, | 7 |
| Karlin NJ, et al. ^33^ | 2012 | USA | retrospective cohort | 1999-2008 | 4.5 | 1,151 | hospital based | medical | 72 in DM, 68 in No-DM | age, gender | 7 |
| van de Poll-Franse LV, et al. ^34^ | 2012 | Netherlands | retrospective cohort | 1997-2007 | NA | 6,974 (colon cancer) | population based | medical records | 73 in DM, 68.8 in no-DM | age, gender, socioeconomic status, stage, lymph nodes examined, adjuvant therapy and year of diagnosis | 8 |
| van de Poll-Franse LV, et al. ^34^ | 2012 | Netherlands | retrospective cohort | 1997-2007 | NA | 3,888  （rectal cancer） | population based | medical records | 71.2 in DM, 66.1 in no-DM | age, gender, socioeconomic status, stage, lymph nodes examined, adjuvant therapy and year of diagnosis | 8 |
| Huang CW, et al. ^37^ | 2012 | China | retrospective cohort | 01/2002-12/2008 | 2.67 | 1,197 | hospital based | medical records | 67.63 in DM, 63.11 in No-DM | age, gender, location of cancer, tumor size, BMI, hitology, stage, vascular invasion, CEA, perineurial invasion | 7 |
| Bella F, et al. ^35^ | 2013 | Italy | retrospective cohort | 2003-2005 | NA | 1,039 | population based | medical records | NA | age, gender, stage, subsite, type fo treatment, morphology, grade | 8 |
| Jeon JY, et al. ^24^ | 2013 | South Korea | prospective cohort | 01/1995-12/2007 | NA | 4,131 | hospital based | medical records | 59 | age, gender, BMI, family history of colorectal cancer, TNM stage, adjuvant therapy, the year of surgery | 8 |
| Luo J, et al. ^25^ | 2014 | USA | prospective cohort | 2003-2009 | 3.17 | 61,213 | population based | medical records | NA | age, gender, race, marital status, stage, census tract median income, co-morbidity | 8 |
| Chen KH, et al. ^26^ | 2014 | China-Taiwan | prospective cohort | 01/2004-12/2008 | 4.6 | 6,937 | population based | medical records | 67.3 | age, gender, stage, adjuvant chemotherapy, comorbidities | 9 |
| Bae S, et al. ^27^ | 2015 | Australia | prospective cohort | 01/2000-12/2013 | 3.6 | 1,116 | hospital based | medical | 70.9 | age | 5 |
| Amptoulach S, et al. ^36^ | 2015 | Sweden | retrospective cohort | 2004-2011 | 3.25 | 207 | hospital based | medical | 65 | age, gender, the ASA score, the Charlson commorbidity index, type of resection, | 8 |
| Fransgaard T, et al. ^28^ | 2016 | Denmark | prospective cohort | 01/2003-12/2012 | NA | 30,493 | population based | medical records | 72.3 in DM, 70 in No-DM | age, gender, ASA score, BMI, blood transfusions, smoking, alcohol consumption, elective or emergency surgery, type of cancer, T stage, lymph node status, distant metastasis. | 9 |
| Tan C, et al. ^13^ | 2016 | Japan | retrospective cohort | 1998-2009 | 15.6 | 96,081 | population based | medical records | 61.8 in DM | age, sex, body mass index (BMI), family history of colorectal cancer, smoking habit, drinking habit, physical activity,(sports and walking) and education | 9 |
| Chen Y, et al. ^14^ | 2017 | Asia | retrospective cohort | 1984-2006 | 12.7 | 77,1297 | population based | medical records | 53.9 | age, smoke, BMI, drink | 8 |
